# From sense of place to visualization of place: examining people-place relationships for insight on developing geovisualizations

**DOI:** 10.1016/j.heliyon.2018.e00547

**Published:** 2018-03-01

**Authors:** Robert Newell, Rosaline Canessa

**Affiliations:** Department of Geography, University of Victoria, 3800 Finnerty Rd, Victoria, BC, V8P 5C2, Canada

**Keywords:** Geography, Psychology

## Abstract

Effective resource planning incorporates people-place relationships, allowing these efforts to be inclusive of the different local beliefs, interests, activities and needs. ‘Geovisualizations’ can serve as potentially powerful tools for facilitating ‘place-conscious’ resource planning, as they can be developed with high degrees of realism and accuracy, allowing people to recognize and relate to them as ‘real places’. However, little research has been done on this potential, and the place-based applications of these visual tools are poorly understood. This study takes steps toward addressing this gap by exploring the relationship between sense of place and ‘visualization of place’. Residents of the Capital Regional District of BC, Canada, were surveyed about their relationship with local coastal places, concerns for the coast, and how they mentally visualize these places. Factor analysis identified four sense of place dimensions - nature protection values, community and economic well-being values, place identity and place dependence, and four coastal concerns dimensions - ecological, private opportunities, public space and boating impacts. Visualization data were coded and treated as dependent variables in a series of logistic regressions that used sense of place and coastal concerns dimensions as predictors. Results indicated that different aspects of sense of place and (to a lesser degree) concerns for places influence the types of elements people include in their mental visualization of place. In addition, sense of place influenced the position and perspective people assume in these visualizations. These findings suggest that key visual elements and perspectives speak to different place relationships, which has implications for developing and using geovisualizations in terms of what elements should be included in tools and (if appropriate) depicted as affected by potential management or development scenarios.

## 1 Introduction

It is widely recognized that incorporating people-place relationships into resource planning is important for ensuring that these efforts are inclusive of the different beliefs, interests, activities and needs associated with areas targeted for management (Cheng et al., 2003; Stocker et al., 2012; Thompson and Prokopy, 2016; Yung et al., 2003). The way local stakeholders relate to place reflects their values and ways of life (e.g., Carter et al., 2007; Panelli et al., 2008; Urquhart and Acott, 2014); therefore, cognizance of these relationships allows for more holistic and socio-culturally sensitive resource management approaches (Poe et al., 2014), enabling inclusion and (ideally) collaboration (Williams and Stewart, 1998). However, albeit important, this incorporation can be difficult to achieve in practice. The relationships people form with places comprise complex and integrative phenomena that are influenced by a wide variety of human and environmental factors (Devine-Wright and Howes, 2010; Gosling and Williams, 2010; Vorkinn and Riese, 2001). In turn, place values are nuanced, and attempting to finely characterize the range of different values can be a complicated task (Cheng et al., 2003). Compounding the challenge is the fact that places can have indistinct boundaries that can not always be spatially defined (McLain et al., 2013), which leads to difficulties around determining exactly to which places certain values are associated. These challenges bring to light a need for tools that can capture and convey ‘place’ in a clear manner and provide better understanding of the people-place relationships that are relevant to particular management plans and strategies.

**Fig. 1:**
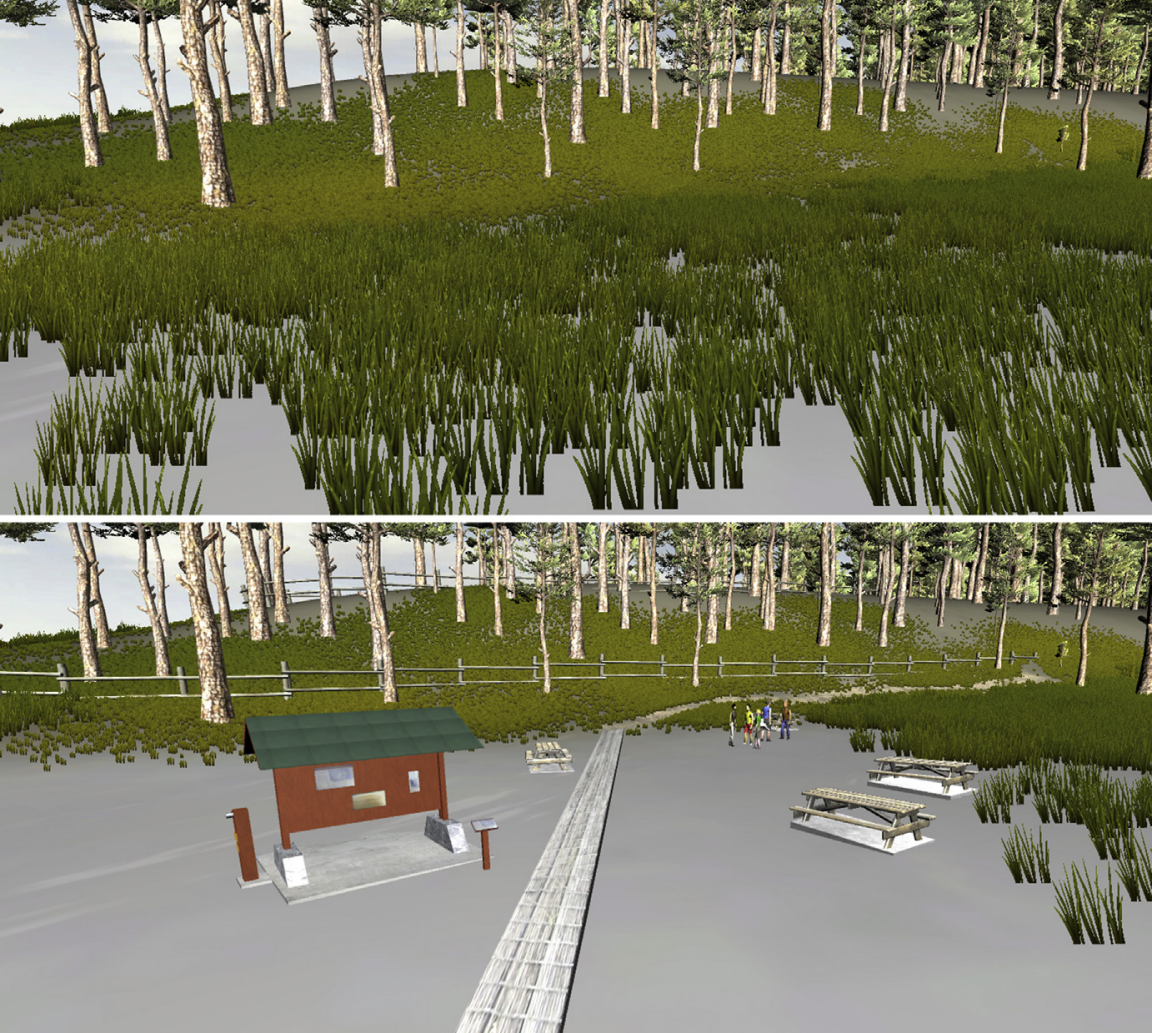
Example of a realistic geovisualization depicting potential modifications to an environment.

Realistic and geographically-accurate representations of real-world places can serve as potentially powerful tools for addressing the aforementioned challenges and allow for resource planning that better incorporates people-place relationships (Newell and Canessa, 2015). Referred to in this study as ‘geovisualizations’ (and elsewhere as ‘landscape visualizations’ (Lewis and Sheppard, 2006) or ‘3D visualizations’ (Grêt-Regamey et al., 2013)), these types of visualizations can provide people with vivid understandings of how they would feel about certain management outcomes or impacts if they transpired in real places (Lewis and Sheppard, 2006; Sheppard et al., 2011). As a result, these tools have been found to elicit strong emotional reactions from users when substantial modifications to familiar places (Fig. 1) are depicted (Salter et al., 2009; Schroth et al., 2009, 2011). Such emotional responses imply that the visuals are connecting with people's ‘sense of place’, i.e., the meanings, values, beliefs and/or feelings people associate with places (Williams and Stewart, 1998), and through this connection, geovisualizations can be used to gain valuable insight on local people-place relationships. For example, these tools can be used to better understand what different people perceive as significant components of a place, how people feel a place should appear and be managed, and what types of development and activities are desirable or undesirable within a place (e.g., Natori and Chenoweth, 2008; Schroth et al., 2011; Tress and Tress, 2003). Subsequently, this insight can inform plans and policy in a manner that ensures local social and cultural values are included within land-use and resource management.

Place-based applications of geovisualizations appear promising; however, this application context is not well understood as geovisualization and sense of place research are very rarely explicitly linked and explored in the same studies. Newell and Canessa (2015) have taken the initial steps toward integrating the two fields of research by developing the theory (through a review-based study) that geovisualizations operate as place-based tools. This study aims to advance this research agenda through empirical work, specifically by pursuing two objectives. The first objective is to establish that a relationship between sense of place and ‘visualization of place’ (i.e., how people mentally visualize place) can be observed using methods that have been developed and well established through previous place research. Once established, the second objective is to explore how different visual elements might relate to different aspects of sense of place and how this insight can be used to inform the development of geovisualizations.

## 2 Background

The current study is part of a larger research project that explores geovisualizations as place-based tools specifically for coastal management and planning. This research follows two review-based studies that (respectively) uncover areas of convergence between place theory and applications of geovisualizations (Newell and Canessa, 2015) and explore place-based considerations around building coastal geovisualizations (Newell and Canessa, 2017a). The findings from this study and the review-based work informed applied research on developing and employing a geovisualization of a particular coastal place located in the Capital Regional District of British Columbia, Canada (Newell et al., 2017a, b). Accordingly, this study focuses on residents living in coastal British Columbia, and it examines the place and visual relationships that said residents form with the local coastal environment.

## 3 Theory

### 3.1 Characterizing and measuring people-place relationships

People-place relationships have been studied through many disciplinary lenses; however, methods for characterizing and measuring these relationships arguably has been most heavily pursued and developed through the disciplines of environmental psychology and human geography (Bott et al., 2003; Lewicka, 2011). This body of work commonly expresses people-place relationships through the concepts of place attachment and sense of place. Albeit closely related, the concepts can differ in connotation and generality. Place attachment specifically refers to the psychological bonding that occurs between people and their environment (Scannell and Gifford, 2010). Sense of place can be considered as broader concept that encompasses place attachment, while also capturing factors such as social and cultural contexts (Campelo et al., 2014).

In studies involving the measurement of place attachment and sense of place, people-place relationships often are characterized as consisting of sub-components or dimensions (e.g., Devine-Wright and Howes, 2010; Kelly and Hosking, 2008; Lee, 2011; Lai and Kreuter, 2012). Williams and Roggenbuck (1989) described place attachment as consisting of two distinct dimensions - place identity and place dependence. Their discussion on place identity referred to Proshansky et al.'s (1983) work, who defined place identity as a sub-structure of self-identity and posited that it comprises the cognitive connection between a person and locality (built from personal meanings, attitudes, feelings and preferences associated with a place). In contrast, place dependence refers to the value a person holds for a place in terms of supporting his or her goals and objectives, and it is associated with the utility of the place such as the resources and recreational opportunities found there (Williams and Roggenbuck, 1989). Williams and Vaske (2003) describe to these two dimensions of place attachment as emotional attachment (place identity) and functional attachment (place dependence).

Jorgensen and Stedman (2001) described sense of place as consisting of three dimensions - place attachment, place identity and place dependence, and they paralleled these dimensions to the three psychological components of attitude - the affective (emotional), the cognitive (meanings and values), and the conative (behavioural inclination). This conceptualization contrasts with Williams and Roggenbuck's (1989) in that it treats place attachment as distinct from identity and dependence, rather than as a ‘second order’ or overarching concept (Kyle et al., 2004). However, regardless of whether place attachment is characterized as first or second order, sense of place can be considered a broader concept. Sense of place encompasses the bonds people form with place, while also capturing other qualities that make a locality a ‘meaningful place’ (Butz and Eyles, 1997; Hay, 1998). Accordingly, some studies on sense of place will examine aspects of place relationships beyond place identity and place dependence, and will explore other meanings and values associated with place. For example, Cross et al.'s (2011) study of landowners residing in Colorado and Wyoming included conservation ethic as a sense of place dimension. As another example, Kaltenborn and Williams's (2002) study of tourists and locals in southern Norway identified values for local culture and history as elements of sense of place. These examples illustrate how sense of place can be considered in terms broader than solely bonds formed with a particular locality, and can capture feelings associated with worldviews and societal values (e.g., environmentalism and care for culture and heritage).

### 3.2 Sense of place and visualization of place

The relevance of place theory to applications of geovisualizations becomes apparent when considering the relationship between sense of place and the way places are visualized. Sense of place is associated with meanings and values people hold for certain localities; therefore, it captures beliefs on how places should be managed or developed (Yung et al., 2003). Accordingly, the way people react to a visual depiction of a management and/or development scenario can be regarded as based on their sense of place and how the visualized elements of a scenario interact with their place meanings and beliefs (Newell and Canessa, 2015). An example of this can been seen through Natori and Chenoweth's (2008) study, in which they prepared a series of landscape images depicting different rice paddy scenarios and presented these images to farmers and naturalists. They found that the farmers showed preference for images of larger paddies with regular patterning (i.e., uniformity among vegetation and paddy shapes), as these images depicted productive easy-to-manage crops. Whereas, the naturalists' preference was toward images of smaller paddies with irregular patterning, as they felt these images conveyed a higher degree of biodiversity. These observations demonstrate how certain visual elements in the landscape imagery (e.g., vegetation, paddy geometry) can interact with the values, interests and meanings associated with place, that is, landscape visualizations can interact with sense of place.

The example above demonstrates how people can consider visual depictions of certain scenarios to be favourable and other depictions as less desirable, which indicates that the different visual representations are either aligning or conflicting with sense of place. Another way of framing this would be that the visual depictions are aligning or conflicting with people's ‘visualization of place’, that is, their perspectives of what places ‘should look like’ and the key visual elements that provide these meanings (e.g., vegetation). Such a proposition is premised on the idea that people hold mental imagery of places, which is a notion developed through earlier work in perceptual geography such as Lynch's (1960) *The Image and the City*. Lynch (1960) employed the term ‘environmental images’ to describe how people form spatial perceptions of urban environments. He noted that these images are comprised of both structural (i.e., physical dimensions) and meaning (i.e., emotional or practical relationships) components due to the fact that they are spatial in nature but also embody personal understandings. Downs and Stea (1977) built on this line of thinking and proposed the term, ‘cognitive mapping’. They described this as the process of a person constructing a mental representation of an environment as he or she navigates through and experiences it, and noted that this representation is inextricably linked to his or her memories and self-identity. Both works employed a more cartographic treatment of imagined geography than what is proposed here with the term, ‘visualization of place’; however, they ultimately support the underlying notion of the visualization of place concept, which is that people develop mental images of places and these images can be based on experiences, meanings and beliefs. Accordingly, it follows that visualization of place is born from sense of place, and the form and character of this visualization is determined through the qualities and strengths of different aspects of sense of place.

**Fig. 2:**
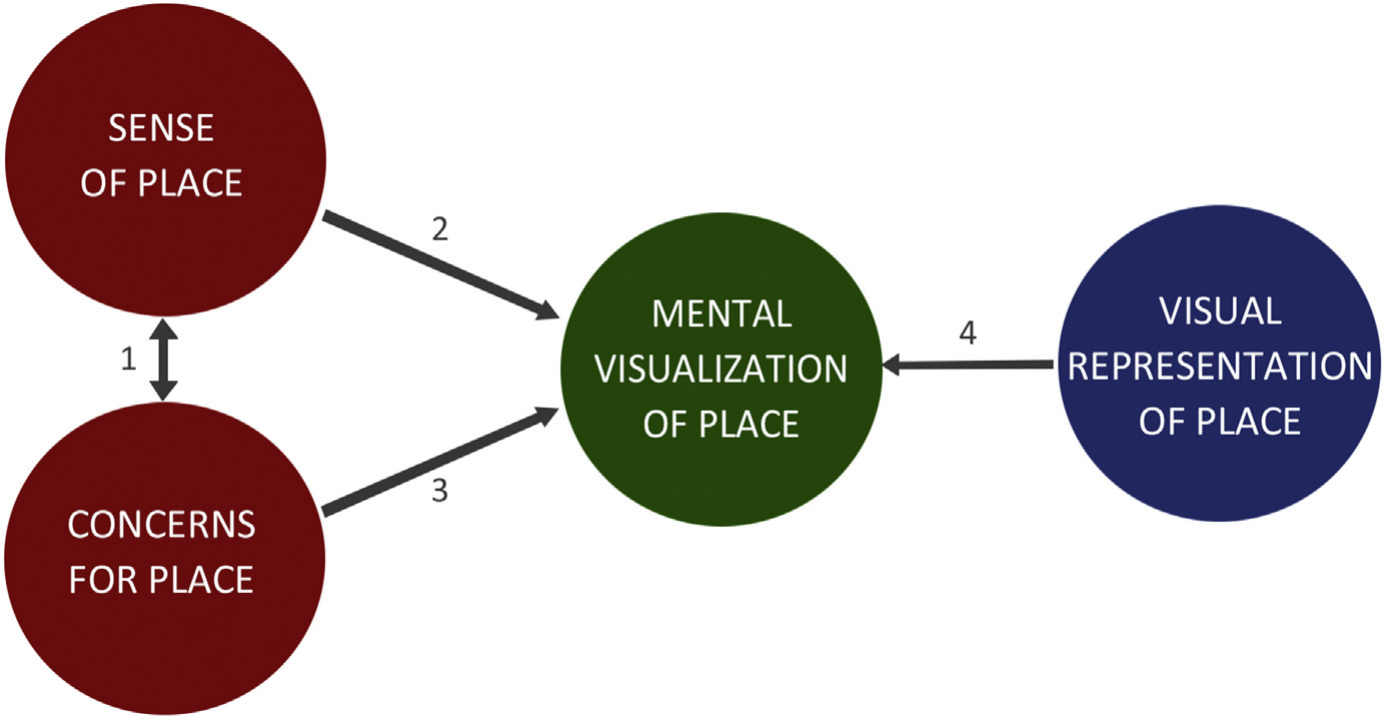
Framework for examining relationship between sense of place and visualization of place. Arrows refer to the following – (1) the meanings and values people hold for places relate to their concerns for these places, (2) sense of place shapes mental visualization of place, (3) place-based concerns influence mental visualization of place, and (4) geovisualizations align or conflict with a person's mental image of place.

The current study explores the relationship between the sense of place and visualization of place for the purposes of advancing a research agenda that integrates place theory and geovisualization studies. There are two ways of approaching this exploration, and these are presented through the analytical framework displayed in Fig. 2. The first and most straightforward approach is to examine sense of place dimensions (see *Characterizing and Measuring people-place relationships*) for their particular influence on visualization of place. The second approach involves using factors related to sense of place as a sort of proxy for place relationships, in particular place-based concerns. Many studies around sense of place and place attachment are conducted in the context of local concerns, and these concerns illuminate relationships between sense of place and reactions to possible alterations to place. For example, Devine-Wright and Howes (2010) described how concerned reactions of Llandudno (Wales, UK) residents toward a proposal for developing windfarms in nearby coastal waters were related to high local place attachment. As another example, Kyle et al.'s (2004) found users of the Appalachian Trail with high place identity expressed more concern for issues such as crowding from people, environmental impacts from trail use and encroachment from human development. In a similar vein, geovisualization studies often elucidate people's concerns for place through how people react to depictions of undesirable management and/or development scenarios. For example, Salter et al. (2009) observed that a visualization depicting increased housing density on Bowen Island (BC, Canada) generated a sense of ‘unease’ in some local residents. This was expressed through concerns around housing encroachment and loss of the ‘character’ of local place, which indicates that the visualization (and depicted housing scenario) was interacting with concerns associated with place. Ultimately, the examples above illustrate that place-based concerns relate to both sense of place and responses to geovisualizations; thus, they present a viable option and alternative route for examining the relationship between sense of place and visualization of place.

The analytical framework displayed in Fig. 2 is useful for outlining the approach of this investigation; however, it is important to recognize that it does not capture all factors pertinent to place relationships and studies. In particular, demographic factors can influence people-place relationships, and accordingly, many studies on sense of place and place attachment include in their analyses variables such as age and gender (e.g., Cross et al., 2011; Kelly and Hosking, 2008; Soini et al., 2012). In addition, relationships with place are shaped by the richness and depth of experiences a person has with their environment (Tuan, 1975); therefore, the location and length of residence also can exert an influence on people-place relationships and some studies will include these types of variables in their analyses (e.g., Brown and Raymond, 2007; Carter et al., 2007; Scannell and Gifford, 2010). In recognition of this previous research, the current study also examines factors such as age, gender, location and length of residency in the analyses, despite these not being featured in Fig. 2.

## 4 Methods

### 4.1 Study area

**Fig. 3:**
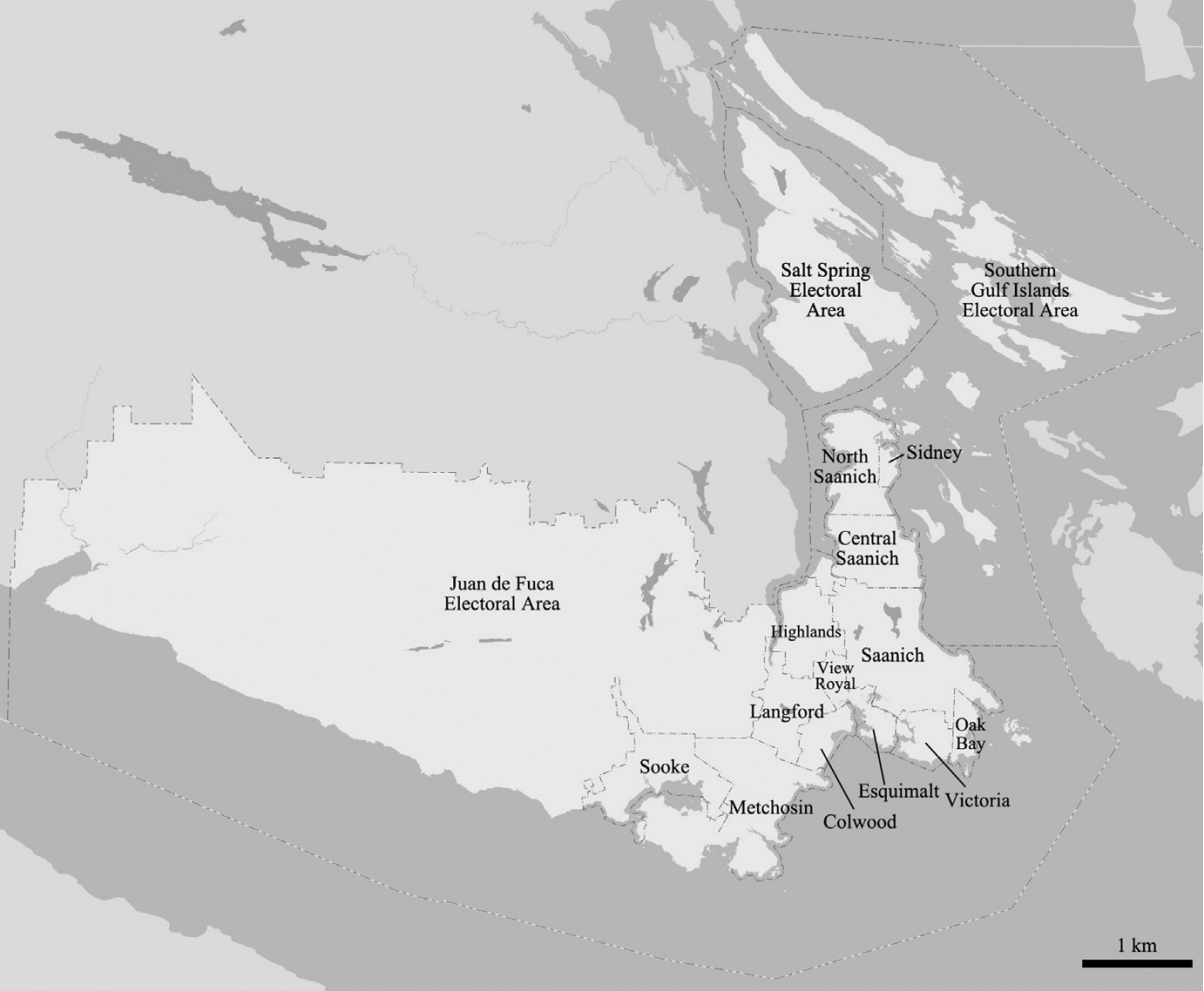
Map of the Capital Regional District in British Columbia. Base map was retrieved from the Capital Regional District Regional Map system.

The study was conducted in the Capital Regional District (CRD) of British Columbia, Canada. The CRD surrounds the provincial capital of Victoria and is located at the southern end of Vancouver Island. It is comprised 13 municipalities, and three electoral areas (Fig. 3). The entire CRD has an area of 2,370 km^2^ (CRD, n.d.); however, this study excludes the Juan de Fuca Electoral Area, resulting in a study area of 868 km^2^.

As this research involved human participants, an ethical review was conducted and approved by the Human Research Ethics Board of the University of Victoria. Survey recipients were provided with a letter of informed consent, which contained information on the research and noted that they are agreeing to participate in the research by filling out and returning the survey. In accordance with the ethical review, details that can be used to identify individuals are not presented in this paper.

### 4.2 Materials

Data were collected through a mail-out survey that was delivered to a random sample of residents throughout the CRD. The survey was tested through a pilot, which was administered to 100 people and resulted in a response rate of 16%. Similar to other survey-based sense of place studies, the survey was developed further based on the results of the pilot (e.g., Soini et al., 2012). The final survey was administered to a random sample of 1,500 people, and the response rate was 18.8% (n = 283). Albeit low, the response rate was comparable to that of other survey-based sense of place studies (e.g., Carter et al., 2007; Moskwa, 2012), as well as was the final sample size (e.g., Jorgensen and Stedman, 2001). The sample might not be entirely representative of the CRD's population; however, the study does not aim to characterize this particular population. Rather, the study is more concerned with collecting a diversity of different coastal place relationships to understand how these can influence mental visualizations of place in different ways, and the sample size is sufficient for these purposes.

The first section of the survey collected demographic data, including gender and age. Gender distribution was relatively equal with 49.1% (n = 139) identifying as male and 48.1% (n = 136) identifying as female (2.8% did not identify their gender). This is comparable to the gender distribution of the CRD as reported through the 2011 Census of Canada (Statistics Canada, 2012), which was reported to be 48.0% males and 52.0% females. Age distribution was skewed to older demographics with 52.7% (n = 149) of respondents over 65, 26.1% (n = 74) of ages 55 to 65, 11% (n = 31) of ages 45 to 54, 5.7% (n = 16) of ages 35 to 44, and 0.4% (n = 1) of ages 19 to 24 (4.1% did not identify their age). Because the 19 to 24 age group contained only one respondent, the data were combined with the 35 to 44 group during the analyses to form an ‘under 45’ group (n = 17). The age distribution of the sample differs from that of the 2011 Census, which reported only 22.6% of the adult (i.e., over 19) population to be 65 and over (Statistics Canada, 2012), whereas this comprised over half of the survey respondents.

As aforementioned, the study is more interested in collecting and analyzing a diversity of place relationships rather than engaging in a specific characterization of the CRD population; therefore, the skewed age distribution was not considered problematic for this study. However, it is worth making mention of the distinct lack of younger respondents, as this could have resulted in missing perspectives and types of place relationships. Age has an effect on sense of place because people develop meanings for and relationships with their local landscape and environment through their life experiences (Hummon, 1992). Accordingly, there can be differences in sense of place between older residents that have had plentiful experiences with a particular locality and younger residents that have not had the same magnitude of place experiences. In effort to capture some of these temporal effects, the survey collected data on how long people have resided in the CRD (as well as their prior place of residency), and lengths of residence was diverse among respondents (M = 32.4, Max = 91, Min = 2). However, such a variable does not fully account for how sense of place is experienced with different age groups, as older residents that have lived in the CRD for shorter periods of time could have formed strong place relationships with other localities/environments (i.e., previous places of residence), whereas this is not necessarily the case with younger groups. Consequently, the lack of younger demographics is considered a limitation of this study, and future research around sense of place and visualization of place should consider targeting certain age groups to more comprehensively explore a diversity of place relationships based on this demographic variable.

In addition to age, gender and length of residence, the first section of the survey also collected data on location of residence. These data were used to develop datasets for two different variables. One such variable involved using addresses to determine how close respondents lived near the shoreline by geocoding addresses in Google Earth (v. 7.1.5.1557), importing into ArcGIS (v. 10.3.1) as point data, and then calculating nearest distances to CRD shoreline (vector data for shoreline was obtained from Statistics Canada, 2011). The other variable involved using addresses to categorize respondents into groups of those who reside on the larger Vancouver Island land mass and those who live in smaller island communities within the surrounding Gulf Islands, which respectively resulted in groups that were 88.4% (n = 251) and 11.6% (n = 33) of the total sample population.

The second part of the survey collected data on how people visualize coastal places and the type of elements they include in their mental imagery. This section was purposely positioned prior to questions concerning sense of place as to not unduly influence the respondents' mental imagery. The pilot survey employed methodology similar to previous studies that examined people's mental models of the environment by asking study participants to sketch pictures related to their models (e.g., Moseley et al., 2009; Shepardson et al., 2007). However, the pilot revealed inconsistencies in how people participated, as some sketched a picture, some provided photographs that they felt represented their mental image, and others wrote a description of their image. This created difficulties for devising a consistent method of identifying and coding elements of the mental visualizations, which was necessary for running regressions and examining the relationships between sense of place data and imaginings of place (see *Results*). Accordingly, the survey was revised, asking participants to describe their image solely through words. This revised methodology drew from Turner et al.'s (2003) study, in which participants were asked to provide written descriptions of a real-world place for the purpose of qualitatively assessing how these descriptions can be interpreted in the context of sense of place. However, unlike Turner et al.'s (2003) work that allowed participants to write descriptions as a free-form narrative, this study asked participants to write five ‘things’ that they visualize (with space to include more, if they wish) in order to collect data that can be easily coded.

Participants were also asked whether they imagined themselves within their mental picture and (if so) how are they positioned and oriented. Such information is important in the context of this study, as the research aims to provide insight on geovisualizations and these forms of visualizations can be developed as egocentric (i.e., viewed from ‘inside’ a virtual environment) or exocentric (i.e., viewed from ‘outside’ the modelled environment) experiences (Orland et al., 2001). Participants were also asked whether they were imagining a specific coastal place or whether they were imagining a place with a ‘typical’ local coastal environment.

The third part of the survey measured sense of place using Likert items, in a similar manner to that performed in previous research on people-place relationships (e.g., Carter et al., 2007; Devine-Wright and Howe, 2010; Jorgensen and Stedman, 2001; Kaltenborn and Williams, 2002; Vaske and Kobrin, 2001). The survey used an even-numbered scale to reduce ambiguity around interpreting neutral responses (Raaijmakers et al., 2000). Responses were on a 6-point scale, and they ranged from ‘strongly disagree’ (i.e., 1) to ‘strongly agree’ (i.e., 6).

Some of the sense of place items were derived from previous research on place attachment (Lai and Kreuter, 2012; Kyle et al., 2004), and these items were typically presented in the first person voice (e.g., “I feel connected to the local coastal environment”). Other items were designed to explore broader aspects of sense of place, such as people's beliefs around how the environment should be treated and managed (Yung et al., 2003). These items were presented in the third person voice (e.g., “It is important that coastal places are protected to maintain the health of local nature”).

The final section of the survey collected data on concerns for local coastal places. Similar to sense of place, the data were collected through Likert items measured on a 6-point scale. Survey participants were presented with a series of events and circumstances that could occur in coastal areas and were asked to rate the extent to which they feel these would be cause for concern if experienced in local coastal places. Responses ranged from ‘not concerned’ (i.e., 1) to ‘a top concern’ (i.e., 6).

## 5 Results

### 5.1 Coding visualization of place

Visualization of place data were coded, allowing for a consistent way of identifying different visual elements among the survey responses. The coding framework was based on a literature review study conducted in previous research that examined how different coastal user interests and needs can influence perceptions of coastal places (Newell and Canessa, 2017a), and it was refined through knowledge and experience the authors gained through building a coastal geovisualization (i.e., what types of visual elements can be modelled, added and manipulated) (Newell et al., 2017a). In total, 86 codes were applied to the data, and in an approach similar to thematic coding (Seidel and Kelle, 1995), this number was reduced to 33 by identifying common themes among the coded elements and grouping them accordingly (Table 1).

**Table 1:** List of types of visual elements and codes used to identify these element types.

Visual element type	Codes
Ocean surface	blue of water (n = 12), reflection on water (n = 7)
Viewshed	ocean view (n = 23), general view (n = 50), mountains (n = 46), sunrise/sunset (n = 8), islands in distance (n = 20), open space (n = 22)
Sky	clear sky (n = 16), clouds (n = 11), general sky (n = 8)
Wildlife	land birds (n = 15), eagles (n = 15), land invertebrates (n = 3), land mammals (n = 4), sea mammals (n = 46), “sea life” (n = 20), fish (n = 16), sea invertebrates (n = 36), general birds (n = 43), land birds (n = 15), seabirds (n = 26), waterfowl (n = 5), shorebirds (n = 7), herons (n = 9), gulls (n = 32)
Terrestrial wildlife	land birds (n = 15), eagles (n = 15), land invertebrates (n = 3), land mammals (n = 4)
Marine wildlife	sea mammals (n = 46), “sea life” (n = 20), fish (n = 16), sea invertebrates (n = 36)
Marine invertebrates	sea invertebrates (n = 36), shells (n = 12)
Domestic animals	cats (n = 2), farm animals (n = 2), dogs (n = 11)
Birds	general birds (n = 43), land birds (n = 15), eagles (n = 15), seabirds (n = 26), waterfowl (n = 5)
Land birds	land birds (n = 15), eagles (n = 15)
Aquatic birds	waterfowl (n = 5), seabirds (n = 26), shorebirds (n = 7), herons (n = 9), gulls (n = 32)
Land plants	forest (n = 122), land plants (n = 38)
Boats	general boats (n = 36), commercial boats (n = 14), ferry (n = 11), fishing boats (n = 7), general large boats (n = 17), recreational boats (n = 24), sailboats (n = 25), general small boats (n = 25)
Large boats	commercial boats (n = 14), ferry (n = 11), fishing boats (n = 7), general large boats (n = 17)
Small boats	recreational boats (n = 24), sailboats (n = 25), general small boats (n = 25)
Marine environment	underwater (n = 3), fish (n = 16), “sea life” (n = 20)
People	people (n = 47), children (n = 11), tourists (n = 7), social interaction (n = 11), playing (n = 9)
Human activity	social interaction (n = 11), playing (n = 9)
Recreation	general recreation (n = 12), walking/hiking (n = 26), playing (n = 9), recreational fishing (n = 20), surfing (n = 5), swimming (n = 17), recreational boats (n = 24)
Land recreation	walking/hiking (n = 26), playing (n = 9)
Ocean recreation	recreational fishing (n = 20), surfing (n = 5), swimming (n = 17), recreational boats (n = 24)
Park structures	park amenities (n = 9), camping (n = 10), beach access (n = 20), parking spaces (n = 3)
Tourism	retail buildings (n = 16), tourists (n = 7)
Development	marina/docks (n = 20), retail buildings (n = 16), houses (n = 35)
Tranquility	little development (n = 27), tranquil/quiet (n = 44)
Environmental quality	clean (n = 48), floating garbage (n = 1), garbage on land (n = 4)
Intertidal area	foreshore (n = 6), tidal pools (n = 14), tidal movement (n = 10)
Beach textures	general beach (n = 102), sand (n = 52), rocks (n = 85)
Woody debris	logs on water (n = 6), logs on beach (n = 25), tree stumps (n = 3)
Algae	“seaweed” (n = 19), kelp (n = 14)
Dynamics	breeze (n = 38), general dynamic (n = 28), tidal movement (n = 10)
Nonvisual senses	warm (n = 11), cold (n = 1), spray/wetness (n = 2), sounds (n = 23), smell (n = 30)
Temperature	warm (n = 11), cold (n = 1)

Number of applications of a code is displayed next to the code in parentheses. Codes that contain the term ‘general’ refer to references that do not specify a particular type of an item. Codes displayed in quotation marks refer to responses that use a particular wording to identify an item.

As discussed above, participants were asked to list at least five items that they ‘see’ within their mental images of coastal place; however, it is important to note that a particular listing could be coded with more than one code. For example, a respondent that lists ‘people socializing with one another’ would be coded with both ‘people’ and ‘social interaction’. In addition, some respondents would list multiple items in one listing (e.g., two or more types of wildlife), and accordingly, these items would be associated with multiple codes.

### 5.2 Exploratory factor analysis

Exploratory factor analyses with varimax rotations were performed on both sense of place and coastal concerns data using SPSS (v. 23), and rotated factor matrices produced from this analysis can be found in Newell and Canessa (2017b). Data were screened to address missing values prior to the analyses, and cases with large amounts of missing values (i.e., many questions that were not answered) were removed. This data screening followed methodology employed by Scannell and Gifford (2010), where cases missing 25% or more data were removed. This resulted in the removal of two cases from the sense of place dataset (i.e., n = 281) and three from coastal concerns dataset (i.e., n = 280). The remaining missing values were imputed prior to conducting the factor analysis using an expected maximization approach, similar to that done in Scannell and Gifford (2010).

Following Raymond et al. (2010), items with factor loadings of 0.4 and greater were considered as potentially part of a factor; however, items that loaded on two or more factors at this strength were excluded unless one loading was significantly higher than the other(s) (i.e., 0.1 or greater). This approach resulted in the removal of one sense of place item and five coastal concerns items, meaning that data from one of the sense of place questions and five of the coastal concerns questions were not included in any of the factors.

The sense of place analysis resulted in the extraction of four factors (Table 2). Factors 1 and 2 relate to participants' beliefs and values concerning coastal places, and they can be respectively described as ‘nature protection values’ (M = 5.60, SD = 0.76) and ‘community and economic well-being values’ (M = 4.73, SD = 1.27). Cronbach's alpha coefficients for these factors were 0.88 (Factor 1) and 0.85 (Factor 2). Factors 3 and 4 roughly correspond to the place attachment dimensions of (respectively) place identity (M = 5.45, SD = 0.92) and place dependence (M = 4.58, SD = 1.24). Cronbach's alpha coefficients for these factors were 0.81 (Factor 3) and 0.82 (Factor 4).

**Table 2:** Rotated factor loadings for factors extracted from sense of place items.

Survey item	Factor			
	1	2	3	4
It is important that local coastal places are protected to maintain their special beauty	0.87			
It is important that coastal places are protected to maintain the health of local nature	0.85			
Coast should be protected so that our families, friends and communities can continue to enjoy these places	0.67			
One of the things I enjoy most about the coast is the local wildlife and nature	0.67			
Coasts are particularly valuable places for supporting wildlife and ecological processes	0.64			
It is important that the coast provides opportunities for local tourism		0.81		
It is important for the local economy that coastal places provide us with resources		0.71		
It is important local coastal places are protected to ensure that our economy can benefit from nature-based		0.69		
It is important that the coast provides the local community with places to interact and socialize		0.59		
It is important that people have the opportunity to live by the coast and adequate housing and services are made available to allow for these opportunities		0.58		
It is important that local parks are established in coastal areas to provide opportunities for outdoor recreation		0.58		
I feel ‘at home’ living near the coast			0.77	
I would miss the coast if I moved away from it			0.69	
I feel connected to the local coastal environment			0.66	
One of the things I enjoy most about living near the coast is the view of the ocean			0.43	
Local coastal areas are some of my favourite places for activities I like to do				0.68
Local coastal areas are my favourite places to spend time with friends and family				0.66
One of the things I enjoy most about local coast places is the recreational opportunities they provide				0.52
I enjoy visiting local coastal areas more than any other type of place				0.50
The relationships I have formed with friends and neighbours in this coastal place (i.e., the CRD) are stronger than relationships I have formed elsewhere				0.50

As seen in Table 2, Factor 4 also contains items related to social bonding, which other research has observed as a place attachment dimension separate from place dependence (Lai and Kreuter, 2012). However, as most of the items in the factor relate more to the functional values of place (e.g., recreational activities), the factor is described as place dependence. In addition, one of the social bonding items alludes to engaging in recreation with other people (i.e., “Local coastal areas are my favourite places to spend time with friends and family”); therefore, it is possible to interpret these results in the context of social opportunities being perceived as a part of the recreational opportunities and functions afforded by coastal places.

The coastal concerns analysis also resulted in the extraction of four factors (Table 3). Factor 1 is described as ‘ecological concerns’ (M = 5.04, SD = 1.24), as it primarily relates to concerns around environmental impacts and loss of biodiversity. Factor 2 is described as ‘private opportunities concerns’ (M = 2.69, SD = 1.61) because it contains items relating to restrictions around both property development and opportunities when on a privately owned vessel. Factor 3 is described as ‘public space concerns’ (M = 4.00, SD = 1.44), as it refers to issues such as access to shoreline and conflicts between public use and protection. Similar to Factor 1, Factor 4 relates to environmental impacts; however, Factor 4 is described as ‘boating impacts concerns’ (M = 3.80, SD = 1.41), as it contains items that specifically relate to impacts from boating and activities that are commonly conducted from boats (e.g., fishing). Cronbach's alpha for Factor 1, 2, 3 and 4 are (respectively) 0.87, 0.81, 0.83 and 0.73.

**Table 3:** Rotated factor loadings for factors extracted from coastal concerns items.

Survey item	Factor			
	1	2	3	4
Declining orca populations	0.89			
Declining salmon populations	0.78			
Loss of wildlife habitat	0.68			
Declining seabird populations	0.68			
Garbage in ocean	0.61			
Loss of eelgrass	0.51			
Coastal erosion and loss of beachfront	0.49			
Garbage on beaches	0.41			
Decreased private property rights in coastal areas		0.82		
Increased restrictions on commercial shoreline development		0.67		
Limited opportunities for recreational boating		0.66		
Limited recreational fishing opportunities		0.64		
Loss of recreational opportunities on the coast			0.80	
Decreased public access to shoreline			0.67	
Loss of tourism opportunities on the coast			0.60	
Conflicts between access/use and protection of the shoreline			0.52	
Impacts from people living aboard boats				0.65
Impacts from recreational boating				0.65
Impacts from fisheries				0.49

### 5.3 Correlation between factors

Correlations among and between sense of place and coastal concerns factors were examined SPSS (v. 23), and output from this analysis can be found in Newell and Canessa (2017b). Sense of place and coastal concern variables were represented through factor scores in the correlation analysis, similar to that done in previous research such as Gosling and Williams (2010) and Scannell and Gifford (2010). This was done to weigh response data according to an item's relevance to a particular factor (Scannell and Gifford, 2010). Factor scores were computed using the regression method, which standardizes the mean score to zero (DiStefano et al., 2009). Cases were removed prior to running the correlation analyses to account for cases of missing data in sense of place and coastal concerns datasets, which resulted in a sample of 278 cases.

Only one statistically significant correlation was observed among sense of place variables. This consisted of a positive correlation between place identity and place dependence (ρ = 0.17, p = 0.01). Analysis of coastal concerns variables also produced only one significant correlation, and this consisted of a very weak negative correlation between private opportunities concerns and public space concerns (ρ = −0.04, p = 0.01).

Several statistically significant correlations were observed between the sense of place and coastal concerns variables. Nature protection values positively correlated with ecological concerns (ρ = 0.47, p < 0.01) and boating impacts concerns (ρ = 0.2, p < 0.01), and negatively correlated with private opportunities concerns (ρ = −0.21, p < 0.01). Community and economic well-being values positively correlated with private opportunities concerns (ρ = 0.26, p < 0.01) and (more strongly) with public space concerns (ρ = 0.46, p < 0.01). Place identity exhibited correlation with only one concerns variable, and this was the ecological concerns variable (ρ = 0.26, p < 0.01). Place dependence exhibited positive (but weak) correlation with all concerns - ecological concerns (ρ = 0.14, p = 0.02), private opportunities concerns (ρ = 0.22, p < 0.01), public space concerns (ρ = 0.15, p = 0.01), and boating impacts concerns (ρ = 0.17, p = 0.01).

### 5.4 Sense of place

The relationship between sense of place and visualization of place was examined through a series of binomial logistic regressions (Equation 1), drawing from methodology used in Cross et al. (2011). Dependent variables consisted of presence/absence of a visual element type (V_i_), coding presence with 1 and absence with 0. Predictors in the regression models consisted of the sense of place variables - nature protection values (P_1_), community and economic well-being values (P_2_), place identity (P_3_) and place dependence (P_4_). Demographic factors were also added to the model, including variables comprising gender (G), age (A_1_, A_2_, A_3_), length of residence in local coastal place (L), distance living from shoreline (D) and whether residence is in a small island (i.e., Gulf Island) community (I). Cases were removed prior to regression analysis due to missing values, and the resultant dataset consisted of 264 cases.

**Equation 1**. Sense of place and visualization of place logistic regression model

V_i_ = β_0_ + β_1_P_1_ + β_2_P_2_ + β_3_P_3_ + β_4_P_4_ + β_5_G + β_6_A_1_ + β_7_A_2_ + β_8_A_3_ + β_6_L + β_6_D + β_6_I

A syntax file was prepared using SPSS (v. 23) to run regressions for all 33 visual element types, and detailed output from this analysis can be found in Newell and Canessa (2017b). The output was examined to identify models that were statistically significant at an alpha of 0.05 (e.g., Cooper et al., 2015). The odds ratios within these models were examined (e.g., Cross et al., 2011) to gain insight on which dimensions of sense of place increased (OR > 1) or decreased (OR < 1) the likelihood of a particular visual element being included in a person's mental visualization of coastal place.

As seen in Table 4, nature protection values increased the likelihood of marine wildlife (OR = 1.82) and wildlife in general (OR = 1.51) being included in visualization of place, while decreasing the likelihood of including non-visual sensory elements, specifically temperature (OR = 0.47). A similar pattern was noticed with place identity, meaning a positive relationship was observed with marine wildlife (OR = 1.77) and negative relationship was observed with temperature (OR = 0.54). Additional relationships were observed with place identity, and these included positive relationships with small boats (OR = 2.18), marine environment elements (OR = 2.75) and ocean recreation (OR = 1.76). Only one relationship was observed with place dependence, in which it increased likelihood of including marine environment elements (OR = 1.71) in visualization of place. No statistically significant relationships were observed with community and economic values in the regression models.

**Table 4:** Results of logistic regressions involving sense of place and visual elements variables.

Visual element (V)	χ^2^	Semi-standardized odds ratios										
		β coefficient										
		Standard error										
		P_1_	P_2_	P_3_	P_4_	G	A_1_	A_2_	A_3_	L	D	I
Wildlife	21.03	1.5**	0.96	1.28	1.16	1.13	1.11	0.99	0.99	0.80	1.09	1.14
		**0.42**	−0.04	0.29	0.17	0.24	0.43	−0.02	−0.02	−0.01	<.001	0.41
		**0.16**	0.15	0.17	0.16	0.28	0.63	0.45	0.32	0.01	<.001	0.48
Marine wildlife	27.22	1.8**	0.99	1.8**	1.15	1.04	1.19	0.98	0.90	0.95	1.36*	1.16
		**0.62**	−0.01	**0.66**	0.16	0.08	0.70	−0.06	−0.24	−.002	<.**01**	0.47
		**0.23**	0.15	**0.23**	0.17	0.29	0.57	0.45	0.34	0.01	<.**001**	0.46
Marine invertebrates	22.36	1.56	0.82	1.41	0.87	1.05	1.36	1.33	1.09	1.16	1.7**	1.33
		0.47	−0.21	0.40	−0.17	0.10	1.30	0.89	0.20	0.01	**0.001**	0.40
		0.31	0.20	0.28	0.23	0.41	0.67	0.55	0.50	0.01	**<.001**	0.71
Aquatic birds	28.80	1.58	0.97	1.29	1.04	1.23	1.12	0.44*	0.89	1.07	0.67*	0.89
		0.48	−0.04	0.30	0.06	0.42	0.47	−**2.53**	−0.25	0.003	<**.001**	−0.37
		0.25	0.17	0.23	0.19	0.32	0.62	**1.05**	0.36	0.01	<.**001**	0.51
Land plants	22.72	1.07	1.00	1.12	1.06	1.11	1.32*	1.38*	1.25	0.87	0.86	1.42*
		0.07	−.003	0.13	0.07	0.21	**1.14**	**0.99**	0.51	−0.01	<.001	**1.10**
		0.14	0.14	0.15	0.15	0.27	**0.57**	**0.43**	0.31	0.01	<.001	**0.46**
Small boats	25.42	1.40	1.43	2.18*	1.08	1.18	0.88	0.66	0.86	1.32	1.14	1.08
		0.35	0.38	**0.90**	0.09	0.33	−0.53	−1.27	−0.33	0.02	<.001	0.25
		0.26	0.21	**0.35**	0.21	0.36	0.84	0.79	0.40	0.01	<.001	0.58
Marine environment	22.24	1.64	1.00	2.75*	1.71*	0.72	0.81	0.96	1.00	1.17	0.96	0.64
		0.52	<.001	**1.17**	**0.62**	−0.65	−0.87	−0.12	−.002	0.01	<.001	−1.41
		0.33	0.22	**0.50**	**0.27**	0.42	1.12	0.65	0.46	0.01	<.001	1.07
Land recreation	20.31	1.53	1.27	0.89	1.27	1.61*	1.11	0.58	0.97	0.83	1.24	0.95
		0.44	0.26	−0.13	0.28	**0.95**	0.42	−1.67	−0.07	−0.01	<.001	−0.17
		0.35	0.24	0.24	0.26	**0.43**	0.70	1.07	0.45	0.01	<.001	0.70
Ocean recreation	21.90	1.19	1.23	1.76*	1.02	1.02	1.00	1.07	0.98	1.47*	1.32	0.91
		0.18	0.22	**0.65**	0.028	0.05	−0.02	0.23	−0.05	**0.02**	<.001	−0.31
		0.23	0.19	**0.29**	0.19	0.34	0.73	0.53	0.40	**0.01**	<.001	0.67
Beach texture	24.04	1.11	0.79	0.80	1.21	1.04	1.23	1.16	1.24	1.14	1.12	0.7**
		0.11	−0.26	−0.26	0.22	0.08	0.87	0.47	0.49	0.01	<.001	−**1.23**
		0.14	0.15	0.16	0.16	0.28	0.63	0.46	0.32	0.01	<.001	**0.45**
Woody debris	26.16	1.47	1.18	1.41	0.91	1.8*	1.53*	1.50*	0.91	1.30	1.16	0.70
		0.40	0.17	0.39	−0.10	**1.14**	**1.73**	**1.25**	−0.23	0.01	<.001	−1.13
		0.37	0.23	0.31	0.24	**0.45**	**1.25**	**0.57**	0.54	0.01	<.001	1.10
Algae	29.24	1.37	1.03	1.24	1.49	1.15	1.65*	1.28	0.91	1.24	1.8**	1.66*
		0.33	0.03	0.25	0.46	0.27	**2.03**	0.76	−0.23	0.01	**0.001**	**1.60**
		0.34	0.24	0.46	0.29	0.46	**0.69**	0.65	0.59	0.01	**<.001**	**0.67**
Temperature	28.16	0.5**	0.73	0.54*	0.60	1.72	1.02	0.79	0.69	0.36*	0.68	0.002
		−**0.80**	−0.34	−**0.71**	−0.58	1.09	0.07	−0.75	−0.83	−**0.05**	<.001	<−10
		**0.27**	0.36	**0.33**	0.37	0.78	1.26	1.26	0.94	**0.03**	<.001	>10

Semi-standardized odds ratios (Menard, 2004), unstandardized β coefficients, and standard errors are displayed on different lines for each variable. Statistically significant results are highlighted in bold, and levels of significance are identified with asterisks next to coefficients (one for p < 0.05 and two for p < 0.01). The gender variable was coded as 0 for male and 1 for female. Age variables were dummy coded with A_1_ representing under 45, A_2_ representing 45 to 54, and A_3_ representing 55 to 65. The over 65 group served as the reference category. Length of time residing in the local area was measured in years, and distance from the coast was measured in metres. Residence in a small island community variable was coded as 1 for those living on small islands and 0 otherwise.

Some significant relationships were observed with demographic variables. Female participants were more likely than male participants to include land recreation (OR = 1.61) and woody debris (OR = 1.77) elements in their visualization of place. People under 45 were more likely to include plants (OR = 1.32), woody debris (OR = 1.53) and algae (OR = 1.65). People aged 45 to 55 exhibited a higher likelihood of including land plants (OR = 1.38), but were less likely to include aquatic birds (OR = 0.44). Location and length of residence also demonstrated an effect on visualization of coastal places. The length of time living in a place increased likelihood of including ocean recreation elements (OR = 1.47), but decreased likelihood of non-visual temperature elements (OR = 0.36). Distance living from the shoreline increased likelihood of including marine wildlife (OR = 1.36), marine invertebrates (OR = 1.74) and algae (OR = 1.80) elements; however, likelihood of including aquatic bird elements (OR = 0.67) decreased with distance. People living in small island communities were less likely to note types of beach textures or sediment (OR = 0.68), but more likely to mention land plants (OR = 1.42) and algae elements (OR = 1.66).

The study only comments on regressions that were found to be statistically significant within the defined model structure, as this is in line with previous research (e.g., Cooper et al., 2015) and also provides a reasonable scope for analysis. However, it is worth noting that some coefficients were found to be significant albeit the overall model was not, and stepwise regressions (using a backward elimination approach) would result in finding new significant models and coefficients. For example, the V_people_ model was not found to be significant at α = 0.05 (χ^2^ = 18.61, p = 0.07), but the place dependence coefficient was significant and positively associated with people visual elements (OR = 1.60, p = 0.01). Future work might wish to take an approach where models are refined to generate more insights on the relationships, but this is out the scope of the current study.

### 5.5 Coastal concerns

Regressions were also conducted between concerns for coastal places and visualization of place (Equation 2). The model assumed a similar format to that of Eq. (1), with the only differences being that ecological concerns (C_1_), private opportunities concerns (C_2_), public space concerns (C_3_) and boating impacts concerns (C_4_) were included, but sense of place variables were not. Coastal concerns and sense of place variables were not included in the same model to reduce multicollinearity effects.

**Equation 2**. Concerns for coastal place and visualization of place logistic regression model

VEC_i_ = β_0_ + β_1_C_1_ + β_2_C_2_ + β_3_C_3_ + β_4_C_4_ + β_5_G + β_6_A_1_ + β_7_A_2_ + β_8_A_3_ + β_6_L + β_6_D + β_6_I

The concerns models were run for all 33 visual element types using an SPSS (v. 23) syntax file, and detailed output from this analysis can be found in Newell and Canessa (2017b). As done with sense of place, only statistically significant models were examined, and fewer models were found to be statistically significant in the coastal concerns analyses (Table 5). Unlike sense of place, no evidence was found for statistically significant coastal concerns regressions that feature land recreation, beach texture and temperature elements as dependent variables.

**Table 5:** Results of logistic regressions involving coastal concerns and visual elements variables.

Visual element (V)	χ^2^	Semi-standardized odds ratios										
		β coefficient										
		Standard error										
		C_1_	C_2_	C_3_	C_4_	G	A_1_	A_2_	A_3_	L	D	I
Wildlife	25.03	1.7**	1.07	0.97	1.20	1.03	1.08	1.04	0.97	0.85	1.10	1.19
		**0.59**	0.08	−0.03	0.21	0.11	0.31	0.11	−0.06	−0.01	<.001	0.542
		**0.16**	0.16	0.16	0.17	0.29	0.65	0.46	0.33	0.01	<.001	0.50
Marine wildlife	27.15	2.1**	0.97	0.98	1.19	0.95	1.15	1.05	0.91	1.09	1.31	1.18
		**0.76**	−0.03	−0.02	0.20	−0.10	0.58	0.17	−0.21	0.004	<.001	0.51
		**0.20**	0.15	0.16	0.18	0.30	0.57	0.44	0.34	0.01	<.001	0.48
Marine invertebrates	22.92	1.57	0.82	1.06	1.38	0.91	1.40	1.45*	1.10	1.28	1.58*	1.19
		0.48	−0.23	0.06	0.38	−0.19	1.36	**1.16**	0.22	0.01	**<.001**	0.54
		0.27	0.23	0.23	0.26	0.42	1.16	**0.55**	0.50	0.01	**<.001**	0.73
Aquatic birds	28.63	1.45	0.91	1.05	1.25	1.15	1.14	0.47*	0.91	1.15	0.64*	0.92
		0.40	−0.10	0.05	0.26	0.27	0.54	−**2.35**	−0.21	0.01	**<.001**	−0.27
		0.21	0.17	0.18	0.20	0.34	0.64	**1.05**	0.36	0.01	**<.001**	0.52
Land plants	21.94	1.04	1.09	0.99	0.96	1.15	1.31	1.38*	1.24	0.88	0.86	1.39*
		0.04	0.09	−0.01	−0.05	0.28	1.09	**1.00**	0.49	−0.01	<.001	**1.04**
		0.15	0.15	0.15	0.16	0.28	0.58	**0.43**	0.31	0.01	<.001	**0.46**
Small boats	19.91	1.59*	1.10	0.95	0.73	1.29	0.81	0.65	0.86	1.52*	1.18	0.99
		**0.50**	0.11	−0.05	−0.37	0.51	−0.85	−1.33	−0.35	**0.02**	<.001	−0.02
		**0.23**	0.19	0.20	0.22	0.37	0.84	0.79	0.40	**0.01**	<.001	0.59
Marine environment	21.78	2.6**	0.84	1.35	1.45	0.66	0.87	1.06	1.06	1.38	0.86	0.70
		**1.01**	−0.19	0.34	0.43	−0.82	−0.54	0.18	0.13	0.02	<.001	−1.14
		**0.36**	0.34	0.24	0.28	0.43	1.13	0.64	0.48	0.01	<.001	1.09
Ocean recreation	19.95	1.18	0.81	0.99	0.70*	1.14	0.93	1.05	0.95	1.6**	1.33	0.81
		0.17	−0.23	−0.02	**−0.41**	0.26	−0.29	0.14	−0.12	**0.03**	<.001	−0.67
		0.19	0.19	0.19	**0.21**	0.35	0.75	0.52	0.40	**0.01**	<.001	0.68
Woody debris	30.58	1.12	0.66	1.36	0.74	1.9**	1.52*	1.55*	0.89	1.44	1.05	0.65
		0.12	−0.45	0.34	−0.35	**1.32**	**1.70**	**1.37**	−0.26	0.02	<.001	−1.38
		0.25	0.25	0.24	0.27	**0.47**	**0.75**	**0.57**	0.55	0.01	<.001	1.13
Algae	24.78	1.06	1.10	1.01	1.17	1.24	1.7**	1.34	0.95	1.25	1.80*	1.67*
		0.06	0.11	0.01	0.18	0.43	**2.16**	0.91	−0.12	0.01	**0.001**	**1.62**
		0.25	0.24	0.26	0.28	0.47	**0.72**	0.63	0.59	0.01	**<.001**	**0.68**

Results are organized in the same manner as Table 4 with semi-standardized odds ratios, unstandardized β coefficients, and standard errors displayed on different lines for each variable. Statistically significant results are highlighted in bold, and levels of significance are identified with asterisks next to coefficients (one for p < 0.05 and two for p < 0.01).

Similar to nature protection values, ecological concerns increased the likelihood of including marine wildlife (OR = 2.05) and wildlife in general (OR = 1.73) in visualization of place. Ecological concerns also held a positive relationship with small boats (OR = 1.59) and marine environment (OR = 2.58) elements. Only one other concerns dimension exhibited a statistically significant relationship, and this was concerns around boating impacts, which held a negative relationship with ocean recreation elements (OR = 0.70).

Demographic variables displayed similar relationships to visual elements in the concerns regressions as they did in the sense of place regressions. Female participants were more likely to include woody debris elements (OR = 1.93), people under 45 were more likely to include algae (OR = 1.70) and woody debris (OR = 1.52), and people aged 45 to 54 had a higher likelihood of including land plants (OR = 1.38) but were less likely to include aquatic birds (OR = 0.47). Location and length of residence variables in coastal concern models also exhibited a similar pattern to that of the sense of place models. Length of time living in a place increased likelihood of including ocean recreation elements (OR = 1.62). Distance from shoreline increased inclusion of marine invertebrates (OR = 1.58) and algae (OR = 1.71), but decreased aquatic birds (OR = 0.67). Furthermore, similar to sense of place, coastal concerns models showed that people living in small island communities were more likely to include land plant (OR = 1.39) and algae (OR = 1.67) elements in their visualization of place.

A few differences were observed between coastal concerns and sense of place models. In the concerns models, age group 45 to 54 were more likely to include marine invertebrate elements (OR = 1.45) and length of residency positively correlated with inclusion of small boats, whereas neither of these relationships were observed as significant with sense of place. Conversely, as noted above, sense of place regressions produced significant relationships involving the under 45 age group and land plants, as well as distance from shoreline and marine wildlife elements; however, these relationships were not found to be significant in coastal concerns regressions.

### 5.6 Location and orientation of mental imagery

Binomial logistic regressions were also conducted on responses to questions around whether people pictured themselves within their visualizations of place (Table 6). When regressing these responses on sense of place and coastal concern predictors, neither model was statistically significant. However, relationships were discovered when further examining the responses of the people that indicated that they were present in their mental image and then classifying the positions where these people located/imagined themselves. Ecological values negatively correlated with people noting they pictured themselves on private property (OR = 0.46) and positively correlated with people noting they are looking seaward in their image (OR = 1.43). In addition, likelihood of people envisioning themselves on a boat in their mental image increased with place dependence (OR = 2.17).

**Table 6:** Logistic regressions on environment and orientation of people's images of coastal place.

	χ^2^	Semi-standardized odds ratios										
		β coefficient										
		Standard error										
		P_1_/C_1_	P_2_/C_2_	P_3_/C_3_	P_4_/C_4_	G	A_1_	A_2_	A_3_	L	D	I
**Sense of place models**												
On private property	34.49	0.5**	0.88	1.15	0.69	1.79	0.01	0.003	0.57	0.58	0.22*	1.04
		**−0.82**	−0.14	0.16	−0.43	1.17	<−10	<−10	−1.26	−0.03	**−.001**	0.12
		**0.24**	0.27	0.35	0.34	0.71	>10	>10	0.84	0.02	**0.001**	0.73
On a boat	21.52	0.72	1.18	1.05	2.17*	0.76	1.22	0.003	1.34	1.23	0.74	0.92
		−0.34	0.18	0.05	**0.89**	−0.56	0.80	<−10	0.66	0.01	<.001	−0.28
		0.23	0.31	0.31	**0.36**	0.53	0.91	>10	0.53	0.01	<.001	0.83
Facing ocean	25.89	1.43*	1.07	0.97	1.02	1.41*	1.35*	1.01	1.16	1.04	1.18	0.85
		**0.37**	0.08	−0.04	0.02	**0.69**	**1.22**	0.02	0.34	0.002	<.001	−0.50
		**0.18**	0.14	0.16	0.16	**0.27**	**0.59**	0.43	0.31	0.01	<.001	0.46
Real-world place	21.06	1.01	1.23	1.40*	1.33	0.96	1.36	1.06	1.14	1.24	0.69*	1.04
		0.01	0.22	**0.39**	0.33	−0.08	1.26	0.17	0.30	0.01	**<.001**	0.12
		0.16	0.17	**0.17**	0.18	0.33	0.82	0.49	0.39	0.01	**<.001**	0.54
**Coastal concerns models**												
On private property	27.31	0.54	1.38	0.73	1.04	1.76	0.01	0.003	0.74	0.53	0.22*	1.00
		−0.65	0.36	−0.36	0.05	1.13	<−10	<−10	−0.69	−0.03	**−.001**	<.001
		0.36	0.33	0.35	0.35	0.73	>10	>10	0.72	0.02	**0.001**	<.001
Facing ocean	23.81	1.14	0.93	1.23	0.90	1.5**	1.33	1.04	1.13	1.06	1.15	0.84
		0.14	−0.08	0.23	−0.12	**0.78**	1.17	0.13	0.28	0.003	<.001	−0.57
		0.15	0.15	0.16	0.17	**0.28**	0.61	0.43	0.31	0.01	<.001	0.47

P_i_/C_i_ headings refer to either a P_i_ variable in the case of the sense of place models, or C_i_ variable in the case of the coastal concerns models. Results are organized in the same manner as Tables 4 and 5 with semi-standardized odds ratios, unstandardized β coefficients, and standard errors displayed on different lines for each variable. Statistically significant results are highlighted in bold, and levels of significance are identified with asterisks next to coefficients (one for p < 0.05 and two for p < 0.01).

Demographic variables also exhibited relationships with how people positioned themselves within visualizations of coastal place. A higher tendency to imagine oneself looking seaward was found with female respondents in both models (sense of place: OR = 1.41, coastal concerns: OR = 1.47) and with people under 45 in the sense of place model (OR = 1.35). In addition, a negative relationship was found between distance residing from shoreline and tendency to imagine oneself on private property in both models (sense of place: OR = 0.22, coastal concerns: OR = 0.22).

Regressions were also conducted on responses to questions around whether people pictured a specific real-world location; however, only the sense of place model exhibited statistical significance. Within this model, place identity was found to increase likelihood of picturing a specific location (OR = 1.40), and distance living from coast decreased this tendency (OR = 0.69).

## 6 Discussion

The first objective of this study was achieved, as a relationship between sense of place and a person's mental visualization of place was observed. The presence of this relationship is supported by the fact that certain regression models resulted in intuitive (or expected) associations between variables, as this indicates that the findings produced from this novel methodology conceptually ‘make sense’. For example, nature protection values increased likelihood of including wildlife elements within visualization of place, which is to be expected as wildlife is a conspicuous component of the natural world. In addition, ecological concerns were positively correlated to nature protection values, and they also led to inclusion of wildlife elements. These intuitive relationships are perhaps ‘less interesting’ in terms of understanding how sense of place influences visualization of place; however, they are important ‘confirmatory’ findings, which demonstrate that this novel application of methodology has effectively exhibited an underlying relationship between people's sense of place and how they visualize places.

Given that a relationship between sense of place and visualization of place was observed, the study's second objective was pursued, which consisted of exploring the nuances within the relationship and their implications for building geovisualizations. Of particular interest are insights around which visual elements appear significant to certain groups or mindsets, as these can inform where attention should be given when building realistic geovisualizations. As noted by Newell and Canessa (2015, 25) “[r]eal-world environments are highly complex” and “[b]uilders of geovisualizations cannot feasibly capture all the elements and features found within a real-world environment; therefore, these builders need to be selective in what they include”. Studies such as the work done here can help inform what elements are essential inclusions and require attention, particularly when thinking about whether proposed scenarios hold implications for these elements. For example, place identity promoted inclusion of marine wildlife elements, but unlike nature protection values, it did not exhibit a significant relationship with the broader (i.e., ‘general’) wildlife visual element grouping. This suggests that place identity is more specific than nature protection values in how it stimulates visualization of wildlife, and it particularly focuses on ocean animals. Accordingly, coastal geovisualization developers should recognize the importance of the general inclusion of wildlife when working with nature-conscious groups (e.g., environmental NGOs); however, they should also understand the particular significance of marine life elements when collaborating with stakeholders of high place identity, especially when featuring scenarios that might affect these species.

The purpose of the above discussion is to ensure that potential impacts to key visual elements that relate to place-based values are accurately shown, that is, to avoid misleading stakeholders by omission. However, it is also important to recognize that misleading can occur through exaggerating or highlighting impacts in a visualization in a deliberate attempt to evoke emotional responses (Shaw et al., 2009). To avoid such misuse, Sheppard (2001) proposed principles for developing visualizations, one such principle being that they are accurate in terms of actual or expected appearances of places. Given that modelling with complete accuracy (i.e., including everything) is not feasible, the aim here is to ensure (as much as possible) that potential impacts to valued place features are displayed accurately to avoid minimizing or omitting significant consequences associated with enacting a management or development plan. However, it also important to identify key visual elements that speak to place values in order to avoid inaccurately representing or exaggerating impacts to these elements in a manner that unduly evokes emotional responses and sways opinions.

In addition to providing insights on key visual elements, the findings also elucidate how different place relationships can influence perspectives taken in a visualization of place. Sense of place can be textured by the ways in which people interact with their environment and the activities they perform there (Shackeroff et al., 2009), and in the context of coastal places, this can include interactions with marine areas as well as terrestrial (e.g., Moskwa, 2012). The ‘texturing’ translates to forming different visual relationships with the coast and experiencing coastal elements from different perspectives (Newell and Canessa, 2017a). In this study, both place identity and place dependence exhibited positive relationships with marine environment elements, which comprised primarily of items coded as ‘fish’ and ‘sea life’. This finding was complemented with observations of place identity also increasing likelihood of small boat and ocean recreation elements, as such elements relate to activities in where humans might interact with marine life. Contrastingly, place dependence did not exhibit a relationship with any other visual element type; however, it did greatly increase the likelihood of visualizing coastal places from the perspective of one positioned on a boat. This suggests that although both place identity and place dependence might lead to visualizing marine elements, these visualizations coalesce from different ‘angles’. Place identity promotes mental pictures that take the form of scenes where activities and elements that ‘belong’ (i.e., align with place meanings) can be viewed from various points within the scene. Such a notion is consistent with other research that has found certain activities commonly performed within community to form the basis of local place meanings and identities (e.g., Panelli et al., 2008; Urquhart and Acott, 2014). In contrast, place dependence does not appear to be contributing to this type of ‘scene perspective’; rather, it increases tendency for one to position him- or herself on a boat within the activity. This is consistent with the nature of place dependence as it relates to one's functional relationship with place and is often framed in terms of recreational activities (Kyle et al., 2004; Lee, 2011; Williams and Roggenbuck, 1989).

The finding that people's visual relationship with place can take different perspectives has important implications for developing and using geovisualizations as planning tools. As discussed in the *Introduction*, geovisualizations can serve as powerful communication tools that can connect with people's sense of place and allow them to understand on meaningful and emotional levels the potential outcomes of management options or development proposals. Lewis and Sheppard (2006, 308) conducted a study that illustrated this power, where they presented forest management outcomes to local community members of Cheam First Nation (Fraser Valley, BC, Canada) and it was noted that realistic landscape visualizations provided “a way better understanding [that is] almost a feeling”. Their finding demonstrates how geovisualizations can provide vivid understanding of place-based changes; however, it is important to note that they developed their visuals based on viewpoints and perspectives that were identified as familiar by the local community members (Lewis and Sheppard, 2006) and thus the displayed views had personal relevance and meaning to viewers. This suggests that visual perspective played a role in the effectiveness of their landscape visualizations, and more broadly speaking, such perspectives can affect the ability geovisulizations have for connecting with the sense of place of different users. Accordingly, these tools should be equipped with the ability to experience them from multiple perspectives to adequately speak to people with different place relationships. In the case of this research, some coastal users with particularly strong place dependence might ‘imagine’ coastal places from the perspective of being on a boat, and thus coastal geovisualizations should include options to examine scenarios from the boaters' perspective to better connect with the sense of place of these user groups. As noted in *Background*, this study has informed applied research on coastal geovisulizations, and following the findings of the study, an option for teleporting and experiencing scenarios from aboard a boat was built into a case study geovisualization developed through the applied work (Newell et al., 2017b).

The discussion above illustrates how various place relationships can lead to different visual perspectives, and in some cases, these differences might allude to or be reflective of potential user conflicts. This was the case when examining nature protection values and mental visualizations that included private property. In a study of cultural models associated coastal places, Thompson (2007) described a sovereignty model, in which coastal values are heavily associated with privately-owned property, and he noted that this cultural model can potentially conflict with models focused more on ecological values (i.e., the ecological model). Thompson (2007), and later Stocker and Kennedy (2009), explained that these conflicts can arise due to potential impacts private property development can have on ecosystems. The current study found evidence of a potential sovereignty culture existing within the surveyed population, as some respondents identified their visualized perspective as to be from private property and this tendency negatively correlated with distance living from the coast. Coinciding with the sovereignty-ecological conflict described above, the nature protection values sense of place dimension negatively associated with the tendency to visualize the coast from a private property vantage point. If this observation is indeed reflective of the sovereignty-ecological conflict, it further supports the notion that geovisualizations should be developed in a manner that allows for multiple points-of-view (e.g., the ability to experience from within and outside of property) in order to include diverse perspectives and allow differences (and potential conflicts) to be brought to the forefront in collaborative planning sessions.

In addition to generating new insights, the analyses also served to challenge hypotheses around relationships between sense of place and visualization of place, thereby improving understanding on how geovisualizations operate among diverse groups of people. For example, the authors expected that place identity would exhibit a significant relationship with viewshed elements; however, this was not the case. Devine-Wright and Howes (2010) noted that the view of the ocean can form an integral component of local place attachment, and in line with this thinking, the survey item, “One of the things I enjoy most about living near the coast is the view of the ocean”, was found to be associated with the place identity factor (although it was the lowest loading item in the place identity factor). A reasonable hypothesis would be that place identity promotes visualization of viewshed elements, but contrarily, place identity did not appear to increase this tendency or promote mental images of place that assume a ‘looking toward the ocean’ perspective. A potential explanation for this lack of relationship presents itself when noting that survey respondents identified a diversity of viewshed elements (e.g., mountains across water, islands in the distance, sunrise, etc.) and these elements were present in a range of mental pictures of coastal places (i.e., coded in over two fifths of survey responses). These observations indicate that ‘views’ (as components of visualizations) can be composed of a large variety of elements and thusly can speak to a range of place relationships. The implications for geovisualization are that user responses to modelled viewshed components are likely not based one particular aspect of their sense of place, and scenarios that affect these components could elicit responses from a variety of people with different relationships to place.

As aforementioned, coastal concerns were hypothesized to be a potentially viable route for analysis (see Fig. 2); however, the concerns regressions resulted in fewer insights than the sense of place analysis. Concerns regressions consequently did not serve well as an alternative analysis, but were somewhat useful as a complementary piece. For example, boating impact concerns exhibited a negative relationship with ocean recreation elements, which is a relationship that was not observed with any of the sense of place dimensions. Therefore, albeit sense of place is comprised of eco-centric dimensions (i.e., nature protection values), it is perhaps too broad to capture feelings toward specific environmental issues (and the related visual elements), whereas a concerns analysis can uncover some of these nuances.

Places are shaped by our interactions with and activities within an environment, and thus they can be considered fundamentally a function of ‘lived’ experiences (Cresswell, 2009). In concert with this line of thought, the current study found that location and length of residency influenced how people visualize place. A person has more opportunity to engage in local activities the longer they reside in a place, and this study found that such activities can shape visualization of place. For example, length of residency increased likelihood of one including ocean recreation elements in his or her image of coastal place. In a similar vein, one's access to a place is (in part) dependent on how near one resides to the locality, and this proximity also appeared to influence visualization of place within this study. For example, the tendency to include aquatic birds decreased with distance living from the shoreline, suggesting that living nearer to the ocean increases interaction with and salience of these species. These findings bring to light the importance of recognizing that people's day-to-day experiences influence how they visualize the coast, and in terms geovisualization, some visual elements might be more significant to particular users, depending on the location and length of time they have lived within an area.

Some respondents identified non-visual elements in their imaginings of place, which reflects how sense of place can be influenced by a wide range of sensory inputs, including smells, sounds, temperatures, etc. (Tuan, 1975). Such an observation is significant to this research because (as the name suggests) geovisualizations are primarily visual tools, and it is important to understand the limitations of reaching people solely through visual stimuli. This being said, the current study produced relatively few insights around non-visual senses, as only two relationships were observed and these were both negative, i.e., nature protection values and place identity decreased likelihood of respondents including temperature in their mental visualizations. In the case of former, it is possible that nature protection values are inherently visual in the way that they bring to mind a multitude of ecosystem elements that can be experienced through sight (i.e., plants, animals, habitat, etc.). In the case of the latter, place identity also increased tendency to visualize specific coastal places, and these might be places that respondents have experienced frequently in a variety of weathers and temperatures, which would reduce likelihood of identifying either ‘warm’ or ‘cold’ as a particular element of place. However, these interpretations are speculative and require further research to validate.

Although the only statistically significant non-visual element regressions were negative, it is important to recognize that ultimately almost a fifth of respondents brought forward non-visual elements when specifically asked to note what they ‘visualize’ in their ‘mental pictures’ of coastal places. The fact that such visually-oriented terminology was employed in the survey instructions yet a fair number of respondents included items such as sounds, smells and temperatures is a strong indication that people relate to place through senses beyond solely the visual. This suggests that future geovisualization research should explore tools that interact with senses beyond just sight, and work has already been done in this area, such as with Bishop and Stock's (2010) interactive landscape visualization that allows user to locate wind turbines by direction of sound. Therefore, albeit this study did not observe any positive relationships between sense of place dimensions and non-visual elements, this is still an area of interest and should be explored further to better understand how to develop place-based tools that can effectively engage users through multiple senses.

## 7 Conclusion

Appleton et al. (2002, 147) noted that “reality will always exceed our ability to simulate it”, meaning that the world is far too complex to model in its entirety. Consequently, when developing realistic geovisualizations, there are limitations in the number and types of elements that can be included and judgments need to be made around what is excluded. By understanding geovisualizations as tools that interact with people's sense of place, we can engage in studies that can guide these judgements by generating insight on key visual elements and perspectives that speak to different stakeholders. In many ways, this study serves as an invitation for sense of place scholars to collaborate with geovisualization researchers to develop better understanding of the ‘human’ aspects of these tools. This work purposely employed research methods commonly used by environmental psychologists and human geographers that study sense of place in order to illustrate how to bridge disciplines and conduct geovisualization research in the context of place theory. The intention behind using this approach is to encourage future researchers to build upon this work and methodology in order to advance an integrated research agenda. It is through such interdisciplinary approaches that we will be able to develop effective tools for managing our resources in a manner that is socially and culturally conscious, as well as environmentally and economically sound.

## Declarations

### Author contribution statement

Robert Newell: Conceived and designed the experiments; Performed the experiments; Analyzed and interpreted the data; Contributed reagents, materials, analysis tools or data; Wrote the paper.

Rosaline Canessa: Conceived and designed the experiments; Contributed reagents, materials, analysis tools or data; Wrote the paper.

### Funding statement

This work was supported by the Sarah Spencer Foundation, who provided a Sara Spencer Foundation Research Award in Applied Social Sciences, and the Social Sciences and Humanities Research Council (SSHRC), who provided funding for the greater research project entitled “Seascape Visualisation for Marine Conservation Planning and Outreach” (grant number: 435-2013-01948). In addition, SSHRC supported the research by providing the first author with a Joseph-Armand Bombardier Canada Graduate Scholarships Program (Doctoral Scholarships).

### Competing interest statement

The authors declare no conflict of interest.

### Additional information

Data associated with this study has been deposited at Mendeley Data under the accession number https://doi.org/10.17632/mc6d73f9r9.1.
